# Were Multiple Stressors a ‘Perfect Storm’ for Northern Gulf of Mexico Bottlenose Dolphins (*Tursiops truncatus*) in 2011?

**DOI:** 10.1371/journal.pone.0041155

**Published:** 2012-07-18

**Authors:** Ruth H. Carmichael, William M. Graham, Allen Aven, Graham Worthy, Stephan Howden

**Affiliations:** 1 University Programs, Dauphin Island Sea Lab, Dauphin Island, Alabama, United States of America; 2 Department of Marine Sciences, University of South Alabama, Mobile, Alabama, United States of America; 3 Department of Marine Science, University of Southern Mississippi, Stennis Space Center, Mississippi, United States of America; 4 Department of Biology, University of Central Florida, Orlando, Florida, United States of America; University of Hamburg, Germany

## Abstract

An unusual number of near term and neonatal bottlenose dolphin (*Tursiops truncatus*) mortalities occurred in the northern Gulf of Mexico (nGOM) in 2011, during the first calving season after two well documented environmental perturbations; sustained cold weather in 2010 and the Deepwater Horizon oil spill (DWHOS). Preceding the stranding event, large volumes of cold freshwater entered the nGOM due to unusually large snowmelt on the adjacent watershed, providing a third potential stressor. We consider the possibility that this extreme cold and freshwater event contributed to the pattern of perinatal dolphin strandings along the nGOM coast. During the 4-month period starting January 2011, 186 bottlenose dolphins, including 46% perinatal calves (nearly double the percentage for the same time period from 2003–2010) washed ashore from Louisiana to western Florida. Comparison of the frequency distribution of strandings to flow rates and water temperature at a monitoring buoy outside Mobile Bay, Alabama (the 4^th^ largest freshwater drainage in the U.S.) and along the nGOM coast showed that dolphin strandings peaked in Julian weeks 5, 8, and 12 (February and March), following water temperature minima by 2–3 weeks. If dolphin condition was already poor due to depleted food resources, bacterial infection, or other factors, it is plausible that the spring freshet contributed to the timing and location of the unique stranding event in early 2011. These data provide strong observational evidence to assess links between the timing of the DWHOS, other local environmental stressors, and mortality of a top local predator. Targeted analyses of tissues from stranded dolphins will be essential to define a cause of death, and our findings highlight the importance of considering environmental data along with biological samples to interpret stranding patterns during and after an unusual mortality event.

## Introduction

**Figure 1:**
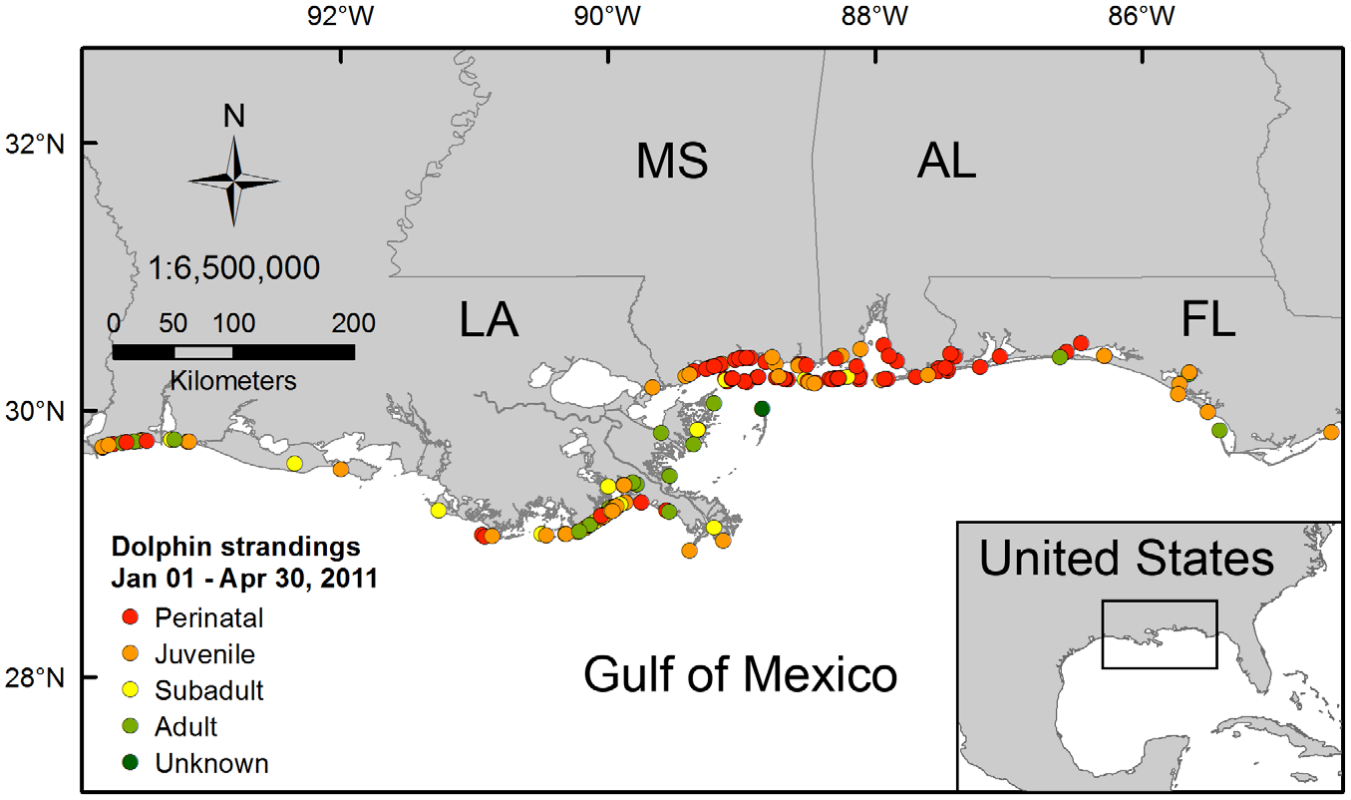
Location of dolphin strandings in the northern Gulf of Mexico, January through April 2011. Dolphin strandings are shown separated by age class (defined in Table 1) for Louisiana (LA), Mississippi (MS), Alabama (AL), and western Florida (FL) [12]. Unknown  =  data not reported.

**Figure 2:**
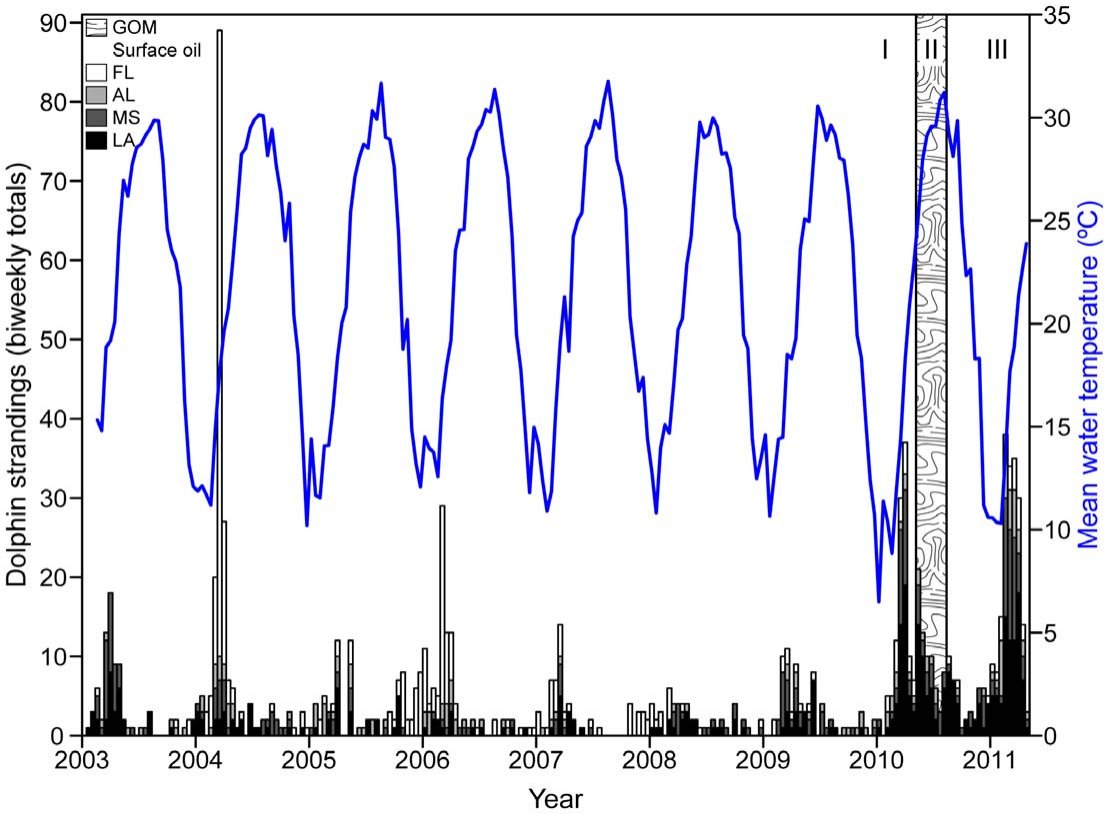
Dolphin strandings compared to water temperature through time. Total biweekly bottlenose dolphin strandings reported in Louisiana (LA), Mississippi (MS), Alabama (AL), and Florida (FL) from January 2003 through April 2011 [12] compared to biweekly mean surface water temperature at Mobile Bay, AL. Potential stressors: I  =  Winter 2010, II  =  DWHOS in the nGOM, III  =  spring freshet.

An unusual number of perinatal (near term to neonatal) bottlenose dolphin (*Tursiops truncatus*) mortalities occurred in waters of the northern Gulf of Mexico (nGOM) from January through April 2011 (Fig. 1). The occurrence of this event early in the first peak calving season after the Deepwater Horizon MC252 oil spill (DWHOS) raised public speculation that the dolphin mortalities were related to toxicity from exposure to oil or dispersant-derived contaminants [1]–[2]. The cause of the unusual mortalities is undetermined, and the National Marine Fisheries Service (NMFS) included them in an ongoing Unusual Mortality Event (UME) that began in February 2010, prior to the DWHOS [3], [4]. The UME was initially prompted by record mortality of primarily adult dolphins, which occurred coincidental with high mortality of other coastal species including finfish, sea turtles, shore birds, and manatees (∼ 6% of the estimated U.S. population of manatees was lost) during sustained cold weather in early 2010 [5], [6]. Dolphins and other coastal species, therefore, experienced at least two potentially stressful events during 2010; the unusually harsh winter conditions followed by the DWHOS (Fig. 2, I and II).

**Figure 3:**
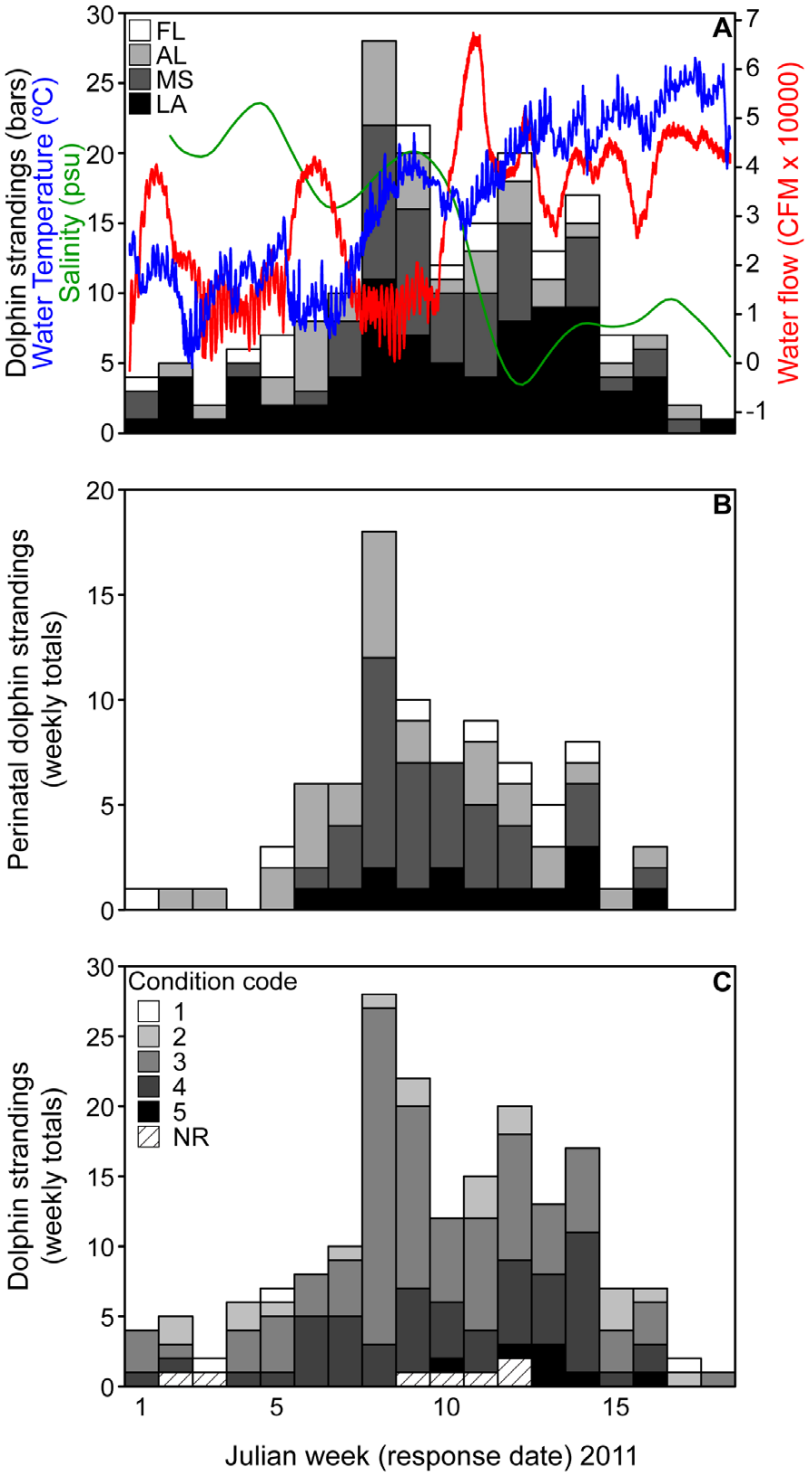
Dolphin strandings in 2011. **A**) Total weekly bottlenose dolphin strandings for each state compared to surface water temperature (30 min intervals), flow (15 min intervals), and salinity (30 min intervals) measured at Mobile Bay, AL. **B**) Weekly perinatal dolphin strandings, separated by state (following the same legend as panel A). **C**) Total weekly bottlenose dolphin strandings, separated by carcass condition on the day of response in 2011. Carcass condition is reported using NMFS standard 5-point code in which 1 is live stranded, 2 is freshly dead, and 5 is most highly decomposed. NR  =  Not reported.

Days prior to the start of the perinatal dolphin stranding event in January 2011, there was a third potential environmental stressor, the rapid entry of large volumes of cold freshwater to near shore coastal waters associated with the melt water from an unusually large winter snowfall in the upper reaches of the Mobile Bay watershed (Fig. 2, III and Fig. 3A). Mobile Bay has the 6^th^ largest watershed and represents the 4^th^ largest freshwater drainage in the U.S. [7]. Although nearshore areas in the nGOM outside Mobile Bay are regularly influenced by this substantial freshwater drainage [8], the watershed had experienced moderate to severe drought conditions for several years [9]. Following a particularly cold winter and the DWHOS in 2010, this subsequent entry of cold freshwater at the nGOM coastline imposed additional stress on the already affected local coastal ecosystem.

Here we propose the possibility that the cold and freshwater event (spring freshet) in early 2011 contributed to the timing and distribution of stranded bottlenose dolphins along the nGOM coast from Louisiana through the Florida panhandle during January - April 2011. To assess this possibility, we compared the frequency distribution of dolphin strandings of different age classes and the reported condition of carcasses to high-frequency collected data for surface water temperature, flow rates, and salinity from monitoring sites at Mobile Bay, Alabama and to water temperature and surface current data for the broader nGOM system from eastern Louisiana through the Florida panhandle.

**Table 1:** Total bottlenose dolphin strandings by state (1 January – 30 April 2011).

	State				
Age class	LA	MS	AL	FL	Total
Perinatal (<115 cm)	14	36	28	8	86
Other juvenile (115–227 cm)	36	13	5	7	61
Subadult (>227–247 cm)	12	5	1	0	18
Adult (>247 cm)	16	0	0	3	19
Not reported	1	1	0	0	2
Total	79	55	34	18	186

LA  =  Louisiana, MS  =  Mississippi, AL  =  Alabama, FL  =  Florida. NMFS independently validated Marine Mammal Health and Stranding Response Program data up to 2008, and data from February 2010 through August 2011 have been audited by NMFS. Data are subject to change prior to NMFS validation.

## Methods

To determine if cold freshwater discharge from the Mobile Bay, Alabama watershed in early 2011 may have contributed to perinatal mortality of dolphins along the nGOM coast during January–April 2011, we compared the frequency distribution of dolphin strandings of different age classes (Table 1) and the reported condition of carcasses to high-frequency collected data for flow rates (15 min intervals), surface water temperature (30 min intervals), and salinity (30 min intervals) from two monitoring sites in the nGOM at Mobile Bay, Alabama. Flow data were collected from the Mt. Vernon (Tensaw River 02471019) U.S. Geological Survey gauge [10], and surface water temperature and salinity were measured at the Dauphin Island environmental monitoring Station, referred to as DPHA1 by the National Data Buoy Center [11]. Environmental data for the Mobile Bay area were smoothed for clarity when compared to dolphin stranding data by using a locally weighted polynomial regression, LOWESS in *R* v.2.13.0.

**Figure 4:**
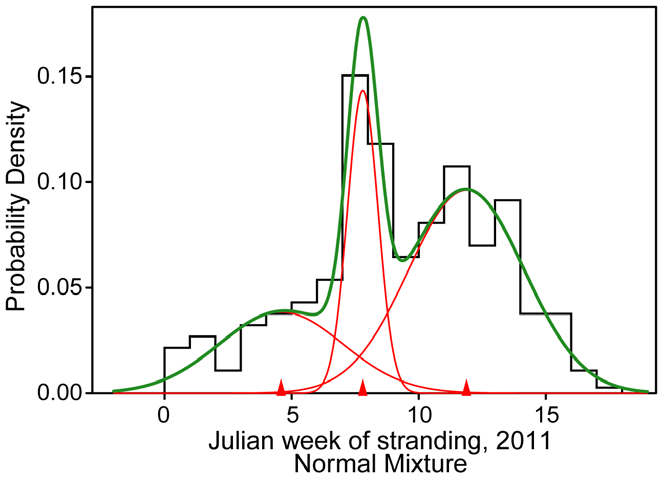
Mixed distribution analysis of 2011 dolphin strandings. The statistically best-fit distribution of total weekly bottlenose dolphin strandings during January – April 2011 [12]. Red lines show the individual components of the best-fit mixture distribution (green), and red triangles indicate the mean of each component distribution. Overlapping distributions were fit iteratively using maximum likelihood of variable combinations (mean, variance, and proportion of each distribution) in the ‘mixdist’ package 0.5–3 in R 2.13.0.

**Table 2:** Comparison of peak dolphin strandings to peak water flow and temperature declines during January – March 2011.

	Julian week of 2011											
	January				February				March			
**Event**	**1**	**2**	**3**	**4**	**5**	**6**	**7**	**8**	**9**	**10**	**11**	**12**
Peak strandings (all)					X			X				X
Peak strandings (perinatal)								X				
Peak water flow	X					X				X	X	
Temperature decline (AL)		X				X				X		
Temperature decline (GOM)						X						

**Figure 5:**
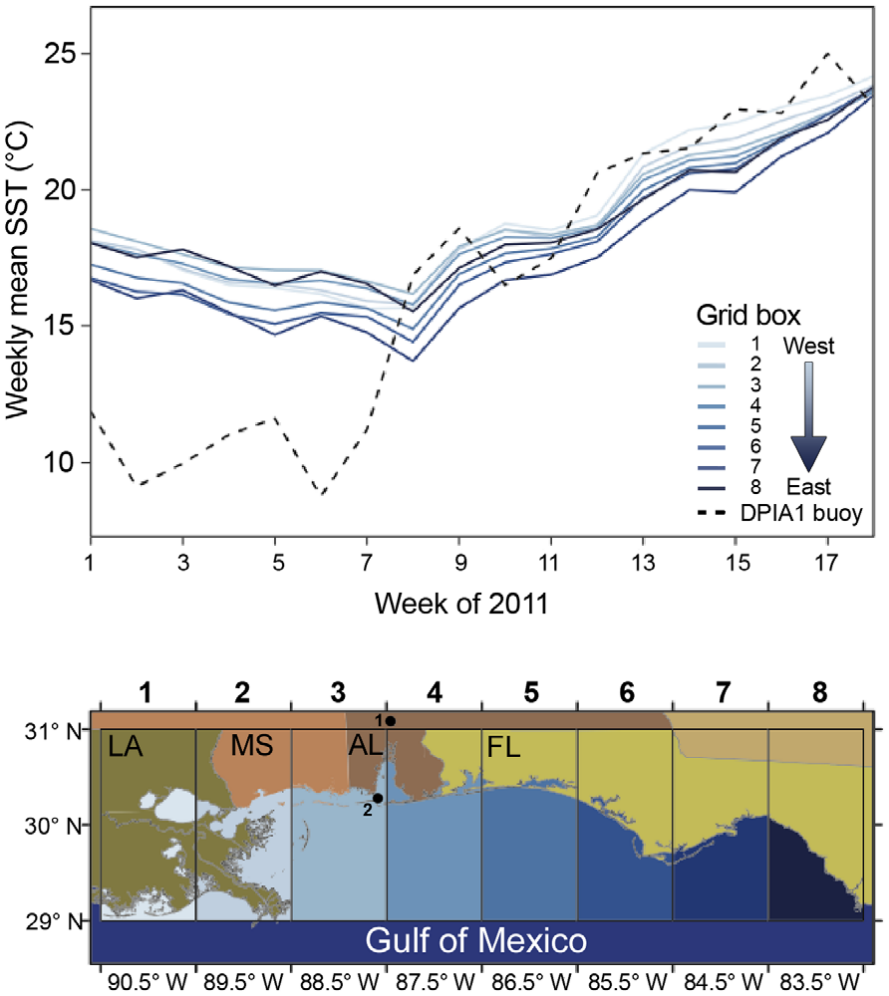
Gulf of Mexico coastal SST in 2011. **A**) Weekly mean sea surface temperature (SST) for near shore areas in the nGOM from 1 January - 30 April 2011. The dashed line is the high-frequency temperature data from Mobile Bay (DPHA1 buoy) shown in Fig. 3A. **B**) 1° longitude×2° latitude grid boxes in which NOAA Weekly Global Sea Surface Temperature SST Model Outputs from OIV2 Optimum Interpolation Analysis (http://www.cpc.ncep.noaa.gov/products/GIS/GIS_DATA/sst_oiv2/index.php) were averaged. Inset dots in **B** indicate sites from which high-frequency local water flow (1) and surface water temperature and salinity (2) data were collected at Mobile Bay, AL. Site 2 is the DPHA1 buoy.

AL  =  sites near Mobile Bay, Alabama during January – March 2011 and correspond to data in Fig. 3A. GOM  =  Gulf-wide (Fig. 5) for the same period.

Dolphin stranding data were obtained from the Southeast U.S. Marine Mammal Stranding Network, reported to the NMFS as of 27 April 2012 [12]. Data used for this study were collected as part of Level A Data (basic stranding data) by the NMFS and included response date (the date that data were collected from the carcass), length (the total straight length from upper jaw to fluke notch), and carcass condition (reported using NMFS standard 5 point code in which a code 1 is live stranded, 2 is freshly dead, and a code 5 is most highly decomposed). Dolphin age classes were assigned using NMFS definitions based on total straight length [13]. Data were collected by trained responders of the Southeast U.S. Marine Mammal Stranding Network and entered into the Marine Mammal Health and Stranding Response Program database hosted by the NMFS. Data were quality checked at the level of data entry by each Stranding Network representative. NMFS has additionally independently validated the Marine Mammal Health and Stranding Response Program database up to 2008, and data from February 2010 through August 2011 have been audited by NMFS. Data are subject to change prior to NMFS validation.

**Figure 6:**
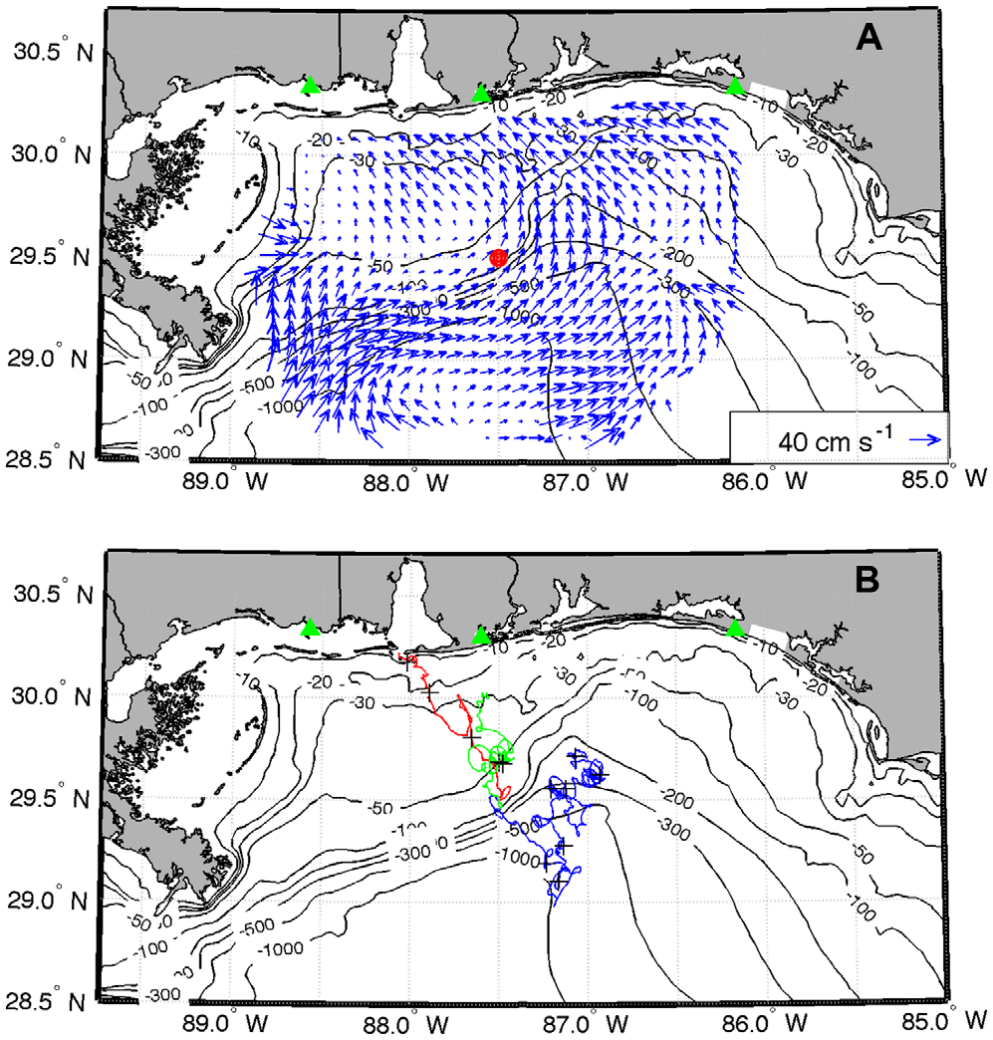
Surface current data measured by CODAR in the Mississippi Bight. **A**) CODAR surface currents measured on 1 January 2011 (blue vectors) at CODAR stations in MS, AL, and FL (green triangles). The red dot indicates the initial position of the pseudo-particle for trajectory runs. **B**) Pseudo-water parcel trajectories initialized on 1 January 2011 (red line), 1 March 2011 (blue line), and 1 April 2011 (green line). For temporal reference, black crosses are plotted every 7 days on each trajectory.

**Figure 7:**
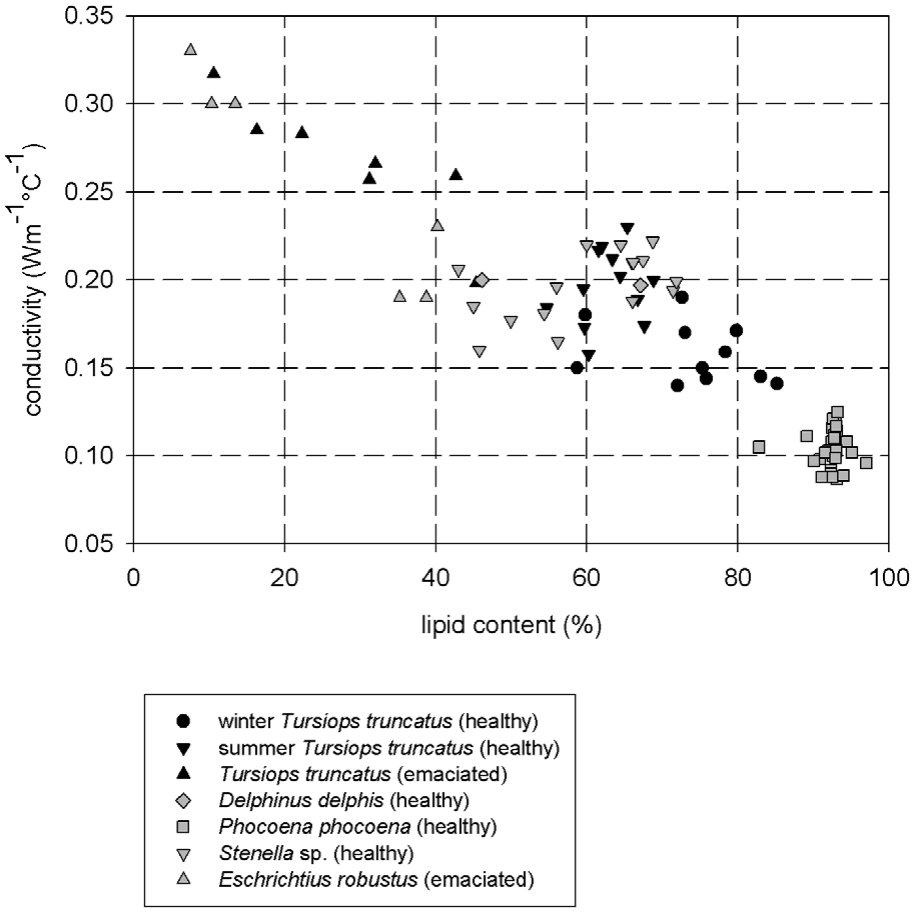
Bottlenose dolphin blubber condition. Conductivity (Wm^−1^°C^−1^) as a function of lipid content (%) of blubber for various species of cetaceans [30, G. W. unpubl. Data]. While healthy bottlenose dolphins showed normal seasonal changes in thermal conductivity (higher in summer when dolphin have lower fat insulation and lower in winter when they have more insulation), emaciated dolphins showed depleted lipid content and, therefore, the highest conductivity values.

To define patterns in bottlenose dolphin strandings during January – April 2011 for comparison to environmental data, we plotted the frequency distribution of dolphin strandings through time and calculated the statistically best-fit normal distributions of total weekly strandings. We grouped dolphin data into one-week intervals because daily comparisons were likely to be less meaningful given delays between death, beaching, carcass discovery and response [14], while two-week intervals or greater could miss variation within the data relative to environmental attributes. Overlapping distributions were fit iteratively using maximum likelihood of variable combinations (mean, variance, and proportion of each distribution) in the ‘mixdist’ package 0.5–3 in R 2.13.0.

To determine the applicability of our detailed environmental data to the broader nGOM system in which dolphins were stranded, we compared our data to publicly available remotely sensed water temperature values and surface current data for near shore waters from eastern Louisiana through the Florida panhandle. We applied NOAA Weekly Global Sea Surface Temperature SST Model Outputs from OIV2 Optimum Interpolation Analysis (http://www.cpc.ncep.noaa.gov/products/GIS/GIS_DATA/sst_oiv2/index.php), averaged for a 1° longitude×2° latitude area. Surface currents in the nGOM were measured by Coastal Ocean Dynamics Applications Radars (CODAR) of the Central Gulf of Mexico Ocean Observing System. Stations in Pascagoula, MS, Orange Beach, AL, and Destin, FL measure surface currents offshore of the 20 m isobath on an hourly basis, with currents averaged over an area of ∼ 36 km^2^. To determine net surface water movement patterns through time, trajectories of pseudo-water parcels were computed by choosing initial points and advecting them with the currents linearly interpolated to the position of the water parcel at each hourly time step. Trajectories were run beginning on days 1 and 15 of each month from January through March, and 1 April.

## Results

During the 4-month period between 1 January and 30 April 2011, 186 bottlenose dolphins, including 86 (46%) perinatal calves (defined as <115 cm), washed ashore from Louisiana to western Florida (Fig. 3A, Table 1). Perinatal strandings during this event were almost 6 times higher than the average number of perinatal strandings in the region since 2003 (15±2) and were nearly double the historical percentage (27±5%) of total strandings. While the majority of carcasses were discovered on the coast of Louisiana and Mississippi, the greatest number of perinatal dolphins stranded on the Mississippi-Alabama coast (Fig. 3B, Table 1). In all, these deaths represent the largest marine mammal mortality event in the nGOM since 2004, when a red tide killed more than 100 bottlenose dolphins off the Florida panhandle [15](Fig. 2).

The temporal frequency of dolphin strandings in early 2011 produced three normal distributions (Fig. 4), with peaks during Julian weeks 5±2, 8±1 and 12±2 (Figs. 3A; *χ* ^2^ = 11.3, df = 10, *p* = 0.33). These time periods correspond to 29 January –4 February, 19–25 February, and 19–25 March 2011 (Fig. 3A, Table 2). Perinatal strandings peaked during week 8 (Fig. 3B, Table 2). Surface water flow peaked during Julian weeks 1, 6, and 10–11, corresponding to temperature drops of 7–10°C in weeks 2, 6, and 10, such that each low temperature event predated carcass discovery by ∼ 2–3 weeks (Fig. 3A, Table 2). Accordingly, the condition of recovered carcasses was also slightly poorer during the later half of the stranding event; the majority of code 4–5 carcasses were discovered after week 10 (Fig. 3C), indicating an offset of at least two weeks between potential cold stress events, mortality, and carcass discovery. This timing is consistent with previous observations in the region [14].

The weekly mean surface water temperatures measured at Mobile Bay in the first few weeks of 2011 were lower than average sea surface temperatures estimated further offshore (Fig. 5). The periodic low temperature events, however, were mirrored in nGOM waters as declines in weeks 2 and 5 at northeastern Gulf of Mexico longitudes and by a decline across all longitudes in week 8 (∼ 25 February), which corresponded to the peak period for all dolphin strandings in the region, shown in Fig. 3A. CODAR current data also showed onshore (north-northwest) movement of surface water from 1 January 2011 (Fig. 6A) through February 2011 (Fig. 6B, red line), corresponding to peak stranding periods in weeks 5 and 8. Surface water trajectories changed to the south in early March (after week 8) and did not return northward until late March (Fig. 6, blue line), corresponding to the third peak of strandings in week 12. Assuming that the currents were coherent from the 20 m isobath to the coastline, these trajectories are consistent with flows that would keep the freshets close to the coast at the peaks times of the strandings. These data suggest pulse temperature depressions were not limited to Mobile Bay, Alabama, and regional water movement favored animals washing ashore in areas immediately offshore from the MS-AL coast where freshwater inputs were most intense.

## Discussion

The relationship between pulsed temperature depressions in the nGOM and local bottlenose dolphin strandings in 2011 is a novel observation. Coastal bottlenose dolphins are exposed to typical fluctuations in water temperature from 5–10°C in winter to as high as 30–35°C in summer. While bottlenose dolphins in good physiological condition are known to tolerate these seasonal temperature ranges, avoid cold areas, and metabolically compensate for heat loss [16]–[18], little is known about dolphin response to dramatic episodic temperature drops like those observed in the nGOM in early 2011. During summer 2011, NOAA undertook a comprehensive health assessment of bottlenose dolphins in Barataria Bay, LA [19]. Preliminary results of the assessment indicated that many of the dolphins analyzed were underweight, anemic, and showed other evidence of poor condition, with almost half of them exhibiting physiological signs of stress [19]. These observations raise the possibility that the Mobile Bay freshet and corresponding regional temperature depressions were contributing stressors to a bottlenose dolphin population that was already in relatively poor physiological condition. Furthermore, the cold freshwater inputs at Mobile Bay occurred during the first of what are typically two broadly seasonal peaks in calving during the year (spring and later summer) [20], consistent with the high frequency of prenatal dolphins among reported strandings.

Based on the timing of events and distribution of strandings, known factors that could have affected dolphin condition prior to the 2011 freshet include: 1) direct exposure to oil or 2) compromised food resources (which could be related to extended colder weather or effects of the DWHOS). Unfortunately, there are few published data to evaluate these possibilities for the period of interest. The sublethal effects of direct oil exposure on dolphins have not been well documented [21], [22], and while perinatal and infant mortality has been associated with exposure to oil and other organic pollutants in some marine mammals, it has not been reported among dolphins [23], [24]. There is better evidence that oil-derived carbon entered the base of the nGOM food web [25], [26] and that prey for dolphins may have been low relative to the number of pregnant females during this time [Smith et al., University of Southern Mississippi, unpubl. Data]. Declines in planktivorous fishes over the shelf in summer and fall 2010 and evidence of genetic and physiological impairment of nearshore fishes support the hypothesis that bottlenose dolphins’ forage base may have been reduced [27, Patterson, University of South Alabama, unpubl. Data]. These data are suggestive that alterations to the local food web from the DWHOS or extreme cold could have moved up the food web to alter body condition of local bottlenose dolphins. As ongoing assessments of coastal food web structure post-DWHOS are published, more data should be available to assess these possibilities.

Previous study provides some corroboration that a combination of exposure to contaminants or sustained cold could lead to poor body condition and mortality of top predators such as dolphins. For example, the combination of high organic contaminant loads and poor nutritional status was associated with high pup mortality, stillbirths and abortions in California sea lions [28]. Interactions between water temperature and prey availability are also thought to affect coastal dolphin distributions, particularly among neonates and other young dolphins [18]. An interaction between reduced food resources and a severe rapid drop in temperature was implicated in the December 1990 death of 26 bottlenose dolphins in Matagorda Bay, TX [29]. Although the 1990 event in Matagorda Bay included only adults, 80% or more of the recovered carcasses showed signs of emaciation [29].

Known changes in the thermal properties of blubber with emaciation may mechanistically link body condition to potential cold-related mortality among dolphins. Lipid content of blubber relates directly to thermal conductivity [30], and importantly, the quality (lipid content) of blubber can be depleted and thermal properties impaired even if the quantity (depth) of blubber appears relatively unchanged [16], [31] and G.W. unpubl. data. Blubber depths of healthy dolphins in nGOM (Texas) waters can range from 16±1 mm in summer to 22±2 mm in winter [32] and G.W. unpubl. data, with corresponding lipid contents of 63±4% and 77±5% and thermal conductivities of 0.20±0.02 Wm^−1^°C^−1^ and 0.16±0.02 Wm^−1^°C^−1^, respectively. These findings are consistent with values in mid-Atlantic coast bottlenose dolphins and healthy free-swimming dolphins in Florida [16], [17], [33]. In emaciated Texas dolphins, however, lipid content dropped to <15% (depth 15 mm – not significantly different from summer depths) with a resultant thermal conductivity of 0.30 Wm^−1^°C^−1^ (Fig. 7) [32]. An animal with the latter blubber quality likely would have difficulty maintaining body temperature under cold conditions. Furthermore, neonate blubber does not appear to show enhanced insulation; prenatal calves and emaciated adults both show reduced blubber thickness, lipid mass, and insulation values compared to healthy juveniles, subadults, and adults [31]. There are no baseline data for blubber condition of dolphins from Louisiana through Alabama, the region of peak strandings in early 2011. The observed poor health of live dolphins from Louisiana examined by NOAA during summer 2011 [19], however, is consistent with these depleted blubber and poor body condition scenarios [29], [31], [32]. Additional data are needed to corroborate these findings for the ongoing UME, but these comparisons demonstrate the potential for factors, such as food quantity and quality, which affect body condition, to interact with cold temperatures and affect mortality.

In addition to nutritive stress related to cold or the DWHOS, a number of possible acute causes of the recent dolphin die-off have been suggested (*e.g.*; disease, toxic algal bloom, or direct exposure to contaminants from the DWHOS) and any of these may have interacted with the spring freshet event. For example, bottlenose dolphins in colder, low salinity waters may be prone to severe skin lesions and physiological stress that make them more susceptible to infection or illness from natural or anthropogenic factors [34]. Accordingly, dolphins assessed in Barataria Bay, LA in summer 2011 had depressed immune systems and generally poor health [19]. Recent analyses indicate that 7 dolphins stranded between 1 January and 30 April 2011 tested positive for *Brucella* spp. (from June 2010 to early May 2012, 25% of dolphins tested were positive or suspected positive for *Brucella* spp.), bacteria commonly found in many marine mammal populations and associated with depressed immune systems, poor body condition, and perinatal mortalities [35]–[38]. It is noteworthy that freshwater discharge, including untreated fish processing waste that is common along the nGOM coast, could be a vector for *Brucella* spp. [39], but remains untested in the region. Future analyses could include examination for freshwater skin lesions and other evidence of freshwater exposure in stranded dolphins relative to the timing of a freshet to better assess this potential contribution to dolphin mortality along the nGOM coast, particularly relative to bacterial infection.

The relative contributions of factors such as a spring freshet, surface current patterns, and modified food webs that indirectly contribute to the timing or distribution of strandings, may be overlooked if not considered alongside potential acute causes of death. Tissue analyses ultimately conducted on field samples from stranded dolphins will be invaluable to align with environmental observations. In these cases, typical measures of acute toxicity or dolphin body condition (blubber thickness, length-weight relationships, and stomach contents) may not be sufficient. Lipid content in relatively fresh blubber, for example, will help better define blubber condition in terms of insulative quality that could link nutritive deficiency and cold-stress to mortality. Analyses of organic carbon, nitrogen, and sulfur stable isotope ratios in recent and archived tissues could help define current and past dietary sources and determine if the stranded dolphins were primarily inshore animals [40], [41] that would have been more likely to encounter the near shore freshwater plume. Newer isotope methods could also help identify or rule out direct oil exposure as well as consumption of oil-contaminated food sources [42]. Importantly, there is a limited window of opportunity to collect the data needed to decipher the driving factors behind any UME, particularly related to an oil spill or ephemeral cold temperatures that may go unnoticed [23], [25].

We propose the possibility that an extreme cold and freshwater event centered on the Mobile Bay watershed in early 2011 contributed to the location and frequency distribution of perinatal strandings among bottlenose dolphins along the nGOM coast in early 2011. Our data suggest cold temperatures were not the sole cause of death, but raise the possibility that the Mobile Bay freshet and corresponding regional temperature depressions were a culminating stressor to a bottlenose dolphin population that was already under stress or in relatively poor body condition (such as due to compromised food resources and bacterial infection). These analyses provide insight to define possibilities and thresholds for understanding this and future UMEs. In particular, we provide strong observational evidence to assess links between the timing of the DWHOS, other local environmental stressors, and mortality of a top local predator. We also highlight the importance of considering interaction between physical environmental variables and biological stressors to inform causes of death during and after an unusual mortality event.
